# Ultrasound imaging assessment of the diaphragm and abdominal muscles in people with a recent history of moderate Covid-19 infection and healthy participants: A cross-sectional pilot study

**DOI:** 10.1371/journal.pone.0281098

**Published:** 2023-02-10

**Authors:** Carlos Romero-Morales, Deborah Falla, Daniel Pecos-Martín, Guillermo García-Pérez-de-Sevilla, Paula García-Bermejo, Emmanuel Navarro-Flores, Daniel López-López

**Affiliations:** 1 Faculty of Sport Sciences, Universidad Europea de Madrid, Villaviciosa de Odón, Madrid, Spain; 2 Centre of Precision Rehabilitation for Spinal Pain (CPR Spine), School of Sport, Exercise and Rehabilitation Sciences, College of Life and Environmental Sciences, University of Birmingham, Edgbaston, Birmingham, United Kingdom; 3 Pain and Physiotherapy Research Group, Physiotherapy Department, University of Alcalá, Alcalá de Henares, Spain; 4 Frailty Research Organized Group (FROG), Department of Nursing, Faculty of Nursing and Podiatry, University of Valencia, Valencia, Spain; 5 Research, Health and Podiatry Group, Department of Health Sciences, Faculty of Nursing and Podiatry, Universidade da Coruña, Industrial Campus of Ferrol, A Coruña, Spain; Camilo Jose Cela University: Universidad Camilo Jose Cela, SPAIN

## Abstract

Coronavirus disease (Covid-19) is a highly infectious disease caused by the SARS-CoV-2 virus and is associated with a decrease of respiratory, physical, and psychological function, subsequently affecting quality of life. The aim of the present pilot study was to use ultrasound imaging (USI) to evaluate and compare the thickness of the diaphragm and abdominal muscles between individuals recently diagnosed with moderate Covid-19 infection and healthy individuals. Methods: A cross-sectional observational pilot study was performed. A total sample of 24 participants were recruited from a private medical center (Madrid, Spain): Covid-19 (n = 12) and healthy controls (n = 12). The external oblique (EO), internal oblique (IO), transversus abdominis (TrA), rectus abdominis (RA), interrecti distance (IRD) and diaphragm thickness were assessed using USI during inspiration, expiration and during contraction. Results: USI measurements of the thickness of EO, IO, TrA, RA, IRD and the diaphragm did not differ significantly between groups during inspiration, expiration or during contraction (all P > 0.05). Conclusions: These preliminary results suggest that the morphology of the abdominal muscles and diaphragm is not altered in people with a recent history of moderate Covid-19 infection.

## Introduction

Coronavirus disease (Covid-19) is a highly infectious disease caused by the SARS-CoV-2 virus and is associated with a decrease of respiratory, physical, and psychological function, subsequently affecting quality of life [1]. Approximately half of Covid-19 survivors present with long-term persistent post-acute symptoms [2] such as dyspnea, fatigue, and muscle weakness, sometimes several months after recovery from the infection [3]. The infection may cause muscle damage due to the so-called cytokine storm [4] and those with severe Covid-19 present with higher inflammatory levels [5] and greater muscle weakness [6, 7]. The high prevalence of muscular-related symptoms in patients with Covid-19 could be due to structural alterations in skeletal muscle [8]. Loss of muscle mass could be related to the impairment of respiratory muscle strength, reduced function and a loss of independence [9].

Previous work has shown that patients with severe Covid-19 infection have a 30% reduction in the cross-sectional area of the rectus femoris [10], and knee extensor weakness was observed in 75% of a cohort of post-Covid-19 patients [11]. De Andadre-Junior et al. reported increased echogenicity in the rectus femoris muscle in patients with severe Covid-19, suggesting qualitative differences in muscle tissue such as fibrosis or fat infiltration, which could lead to lower specific tension, and therefore muscle weakness [11].

Respiratory muscles were classified in two main groups: primary, composed by the diaphragm and intercostal muscles which act expanding the chest wall. The sternocleidomastoid, scalenes and triangularis sterni were considered as accessory muscles [12]. The expiration process has no require muscles activity due to the elastic recoil of the chest wall and the lung. However, in forced situations, the abdominal wall muscles could be activated, being accessory respiratory muscles [12]. Muscles of the abdominal wall are arranged in layers consisting of the external oblique (EO), internal oblique (IO), and transversus abdominis (TrA), with the rectus abdominis (RA) in the midline [13]. In healthy people, these muscles work in a coordinated manner with the diaphragm, lumbar multifidus, and pelvic floor muscles to provide support to the spine [14]. The TrA muscle is considered to be an accessory muscle for respiration under normal circumstances. A disruption of the control of these muscles can occur in conditions such as low back pain, respiratory disorders or urinary incontinence [15, 16].

Ultrasound imaging (USI) is a valid, safe, and cost-effective imaging technique which can be used assess the architecture and texture of soft tissues [17, 18]. Because of the depth of the abdominal wall and diaphragm, USI is one of the few non-invasive and reliable tools that can be used to measure the morphological characteristics of these muscles [19]. Various studies have assessed the diaphragm of patients with Covid-19 with USI, as there is a high prevalence of reported ventilator-induced diaphragm dysfunction in hospitalized patients [20]. One study demonstrated that diaphragm thickness was significantly reduced in Covid-19 patients after seven days of intensive care, especially among non-survivors [21]. According to Corradi et al., a thinner diaphragm may contribute to severe respiratory failure in Covid-19 hospitalized patients [22]. Additionally, most hospitalized patients present with long-term sonographic abnormalities of the diaphragm muscle, such as a significant reduction in the diaphragm muscle thickening ratio, compared with non-Covid cohorts [23]. What has not been investigated, is whether there is a change in abdominal muscle thickness in patients recently diagnosed with Covid-19. This is relevant given the contribution of the abdominal muscles to respiration. Thus, the aim of the present study was to evaluate and quantify with USI, the thickness of the EO, IO, TrA, RA and diaphragm between individuals recently diagnosed with moderate Covid-19 and healthy participants. We expected that differences in both abdominal and diaphragm thickness would be evident in those that have experienced a recent moderate Covid-19 infection compared to controls.

## Materials and methods

### Study design

A cross-sectional observational pilot study was performed from April to May 2021. The study was designed and developed based on the Strengthening the Reporting of Observational Studies in Epidemiology (STROBE) guidelines [24].

### Ethical statement

This study received approval from the Research Ethics Committee at the Universidad Europea (Spain). Before the study commenced, all participants signed an informed consent form. The study was conducted in accordance with all current regulations on human experimentation, as well as the Declaration of Helsinki [25].

### Sample

A total sample of 24 participants aged between 18 to 55 years were recruited for this pilot study and consisted of a group with recent moderate Covid-19 infection (n = 12) and a healthy group (n = 12). Participants for the moderate Covid-19 group were recruited from a private medical center and healthy participants were recruited by announcements or email.

For the Covid-19 group, participants were included if they presented with the following inclusion criteria: recent definitive diagnosis of Covid-19, no history of musculoskeletal disorders over the last 6 months and no respiratory disease. All the participants were diagnosed by a medical doctor from the Spanish Sanitary Services based on a positive polymerase chain reaction (PCR) test. The participants presented with symptoms ranging from 15–25 days and were classified as having moderate Covid-19. Moderate Covid-19 implies the presence of fever or respiratory tract symptoms and imaging results confirming signs of pneumonia [26]. Testing of the participants took place as soon as they presented with a negative PCR-test.

The control group consisted of healthy individuals with no history of musculoskeletal disorders over the last 6 months and no respiratory disease. The exclusion criteria for both groups were: participants with any systemic disease, hospitalization, moderate or severe heart disease, abdominal interventions, bone fractures, ischemic or neurodegenerative diseases [27, 28]. In both groups, anyone with a BMI more than 29.9 was excluded.

### Ultrasound imaging

**Fig 1:**
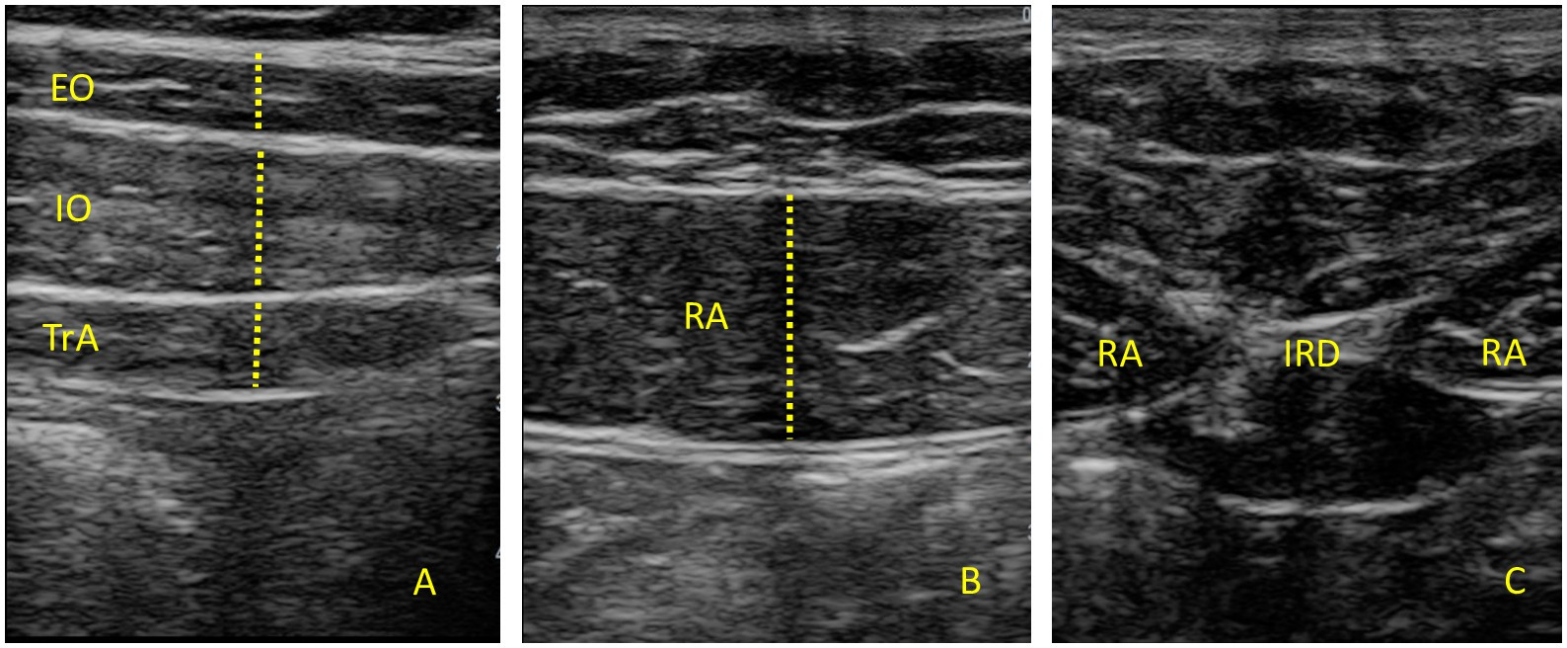
Ultrasonography of the abdominal wall muscles and IRD.

**Fig 2:**
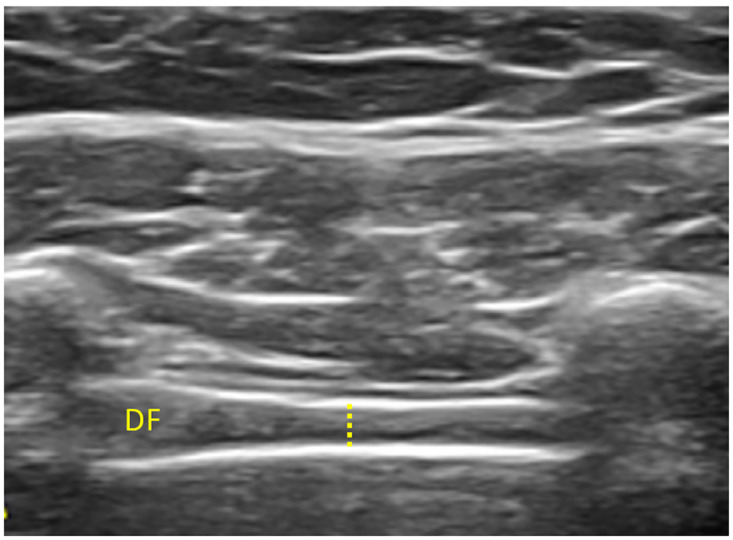
Ultrasonography of the diaphragm muscle.

B-mode USI measurements were performed using a Samsung H40 ultrasound system with a 4–13 MHz linear transducer (38-mm footprint). All the measurements were developed by the same evaluator with more than 10 years of experience in neuromusculoskeletal USI (P.G.B). For assessment of the EO, IO and TrA, the participants were in supine and the transducer was located in the mid-axillary line, between the subcostal line and the iliac crest (Fig 1A) [13]. A 10 MHz frequency, 60-point gain, 60-point dynamic range and 1 foci was used in order to maintain image features constant during image extraction. Muscle thickness was defined as the distance between the inside edges of each muscle border [15]. To assess the muscle at the end of inspiration and expiration, patients were instructed to inhale/exhale normally and hold their breath until the examiner took the images [29]. To assess the contraction time of the abdominal muscles, patients were asked to perform isometric ipsilateral hip flexion whilst lying in supine [30]. For the RA, the transducer was aligned with the umbilicus, and just under the umbilicus for the inter-rectus distance (IRD) evaluation (Fig 1B and 1C). The IRD was determined as the distance between both RA muscles [13]. For the diaphragm, the transducer was located transversely over the lowest intercostal spaced that allowed good visualization. This muscle was recognized by “its typical 3-layered appearance and location deep to the intercostal muscle layer and ribs” (Fig 2) [31]. For all measurements, three images were taken at the end of inspiration, at the end of expiration and during a contraction which was achieved by having the participant perform ipsilateral hip flexion at 45° with full knee extension [32]. The mean of the three measures was calculated for statistical analysis [33]. ImageJ software was used to measure the images offline [34].

### Statistical analysis

Data were analyzed using the statistical program SPSS (SPSS Version 23.0, IBM, Armonk, NY, USA) considering an α error of 0.05, a β error of 0.02, and a confidence interval of 95%. First, the Shapiro–Wilk test was performed to assess the distribution of the data. Second, the descriptive analysis for the total sample and in both groups was carried out. For parametric data, the data are presented as mean and standard deviation (SD) and the Student’s t test was applied whereas for non-parametric data, the data are presented as median and interquartile range (IQR) and the Mann-Whitney U test was employed. In addition, the effect size was estimated with Cohen’s d formula: (M2 − M1)/SD. Given the limited sample size for the present pilot study, a post-hoc power calculation was determined for each variable. For the effect size, d = 0.2 was considered a ‘small’ effect size, 0.5 a ‘medium’ effect size, and 0.8 a ‘large’ effect size.

## Results

**Fig 3:**
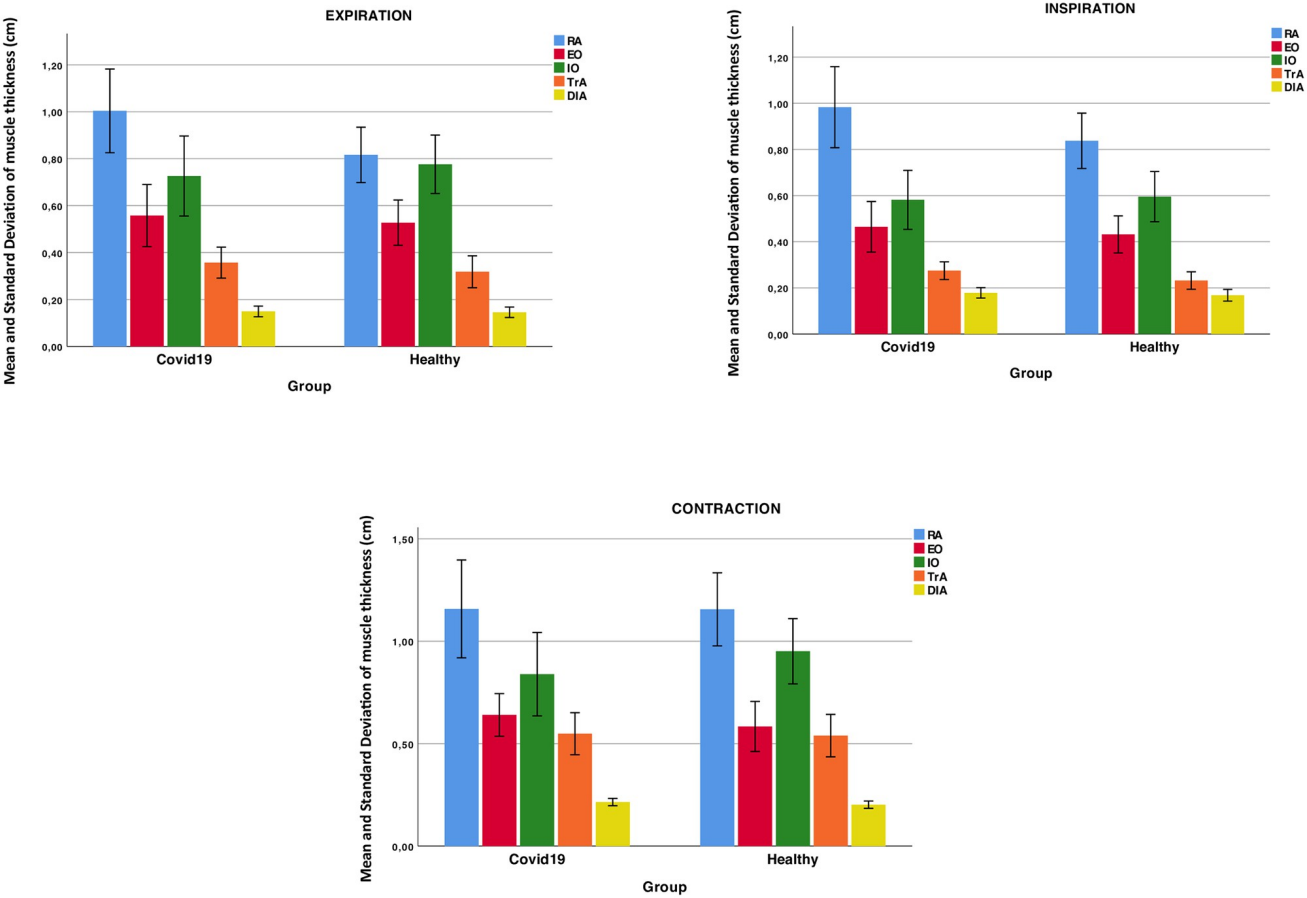
Ultrasonography comparisons between moderate Covid-19 infection and healthy participants.

There was no difference in sociodemographic variables between groups (Table 1). As presented in Table 2, USI measurements of the thickness of EO, IO, TrA, RA, IRD and diaphragm muscles did not differ significantly (all P > 0.05) between groups when measured during inspiration, expiration or during contraction (Fig 3).

**Table 1:** Participant demographic data. Mean ±standard deviation.

Data	Total sample (n = 24)	Covid-19 (n = 12)	Healthy (n = 12)	*P*-value
Age, years	42.04 ± 9.17	42.07 ± 10.57	42.70 ± 7.70	0.984**
Weight, kg	64.47 ± 11.66	66.84 ± 13.25	61.68 ± 9.29	0.290**
Height, m	1.63 ± 0.07	1.62 ± 0.07	1.63 ± 0.13	0.321^‡^
BMI (kg/m^2^)	24.15 ± 4.95	25.46 ± 5.71	22.61 ± 3.62	0.165**

** Student´s t-test for independent samples was performed.

^‡^ Mann-Whitney U test was utilized.

**Table 2:** Ultrasound imaging measurements between groups.

Measurement	Covid-19 (n = 12)	Healthy (n = 12)	95% CI minimum- maximum	*P*-value (Cohen *d* effect size)
**IRD (cm)**				
Insp	1.56 ± 0.75	1.42 ± 0.98	-0.591–0.878	0.689 (0.160)**
Exp	1.48 ± 0.74	1.47 ± 1.02	-0.748–0.755	0.993 (0.011)**
Cont	1.59 ± 0.78	1.36 ± 0.85	-0.458–0.930	0.448 (0.281)**
**RA Thickness (cm** ^**2**^ **)**				
Insp	0.98 ± 0.29	0.83 ± 0.17	-0.063–0.354	0.162 (0.631)**
Exp	0.88 ± 0.48	0.81 ± 0.17	-0.023–0.039	0.079 (0.799)^‡^
Cont	1.15 ± 0.39	1.15 ± 0.26	-0.228–0.293	0.987 (0.001)**
**EO Thickness (cm)**				
Insp	0.46 ± 0.18	0.43 ± 0.11	-0.100–0.165	0.614 (0.201)**
Exp	0.55 ± 0.21	0.52 ± 0.14	-0.129–0.190	0.698 (0.202)**
Cont	0.64 ± 0.17	0.53 ± 0.15	-15.038–5.790	0.445 (0.009)**
**IO Thickness (cm)**				
Insp	0.58 ± 0.21	0.59 ± 0.16	-0.175–0.477	0.860 (0.053)**
Exp	0.72 ± 0.28	0.77 ± 0.18	-0.250–0.149	0.619 (0.212)**
Cont	0.83 ± 0.33	0.95 ± 0.23	-0.362–0.139	0.366 (0.421)**
**TrA Thickness (cm)**				
Insp	0.27 ± 0.06	0.23 ± 0.05	-0.008–0.093	0.097 (0.724)**
Exp	0.33 ± 0.10	0.31 ± 0.10	-0.051–0.128	0.380 (0.400)^‡^
Cont	0.54 ± 0.16	0.53 ± 0.15	-0.129–0.147	0.890 (0.064)**
**Diaphragm(cm)**				
Insp	0.17 ± 0.03	0.16 ± 0.03	-0.021–0.042	0.509 (0.333)**
Exp	0.14 ± 0.03	0.13 ± 0.02	-0.026–0.033	0.799 (0.392)^‡^
Cont	0.21 ± 0.29	0.20 ± 0.26	-0.011–0.036	0.280 (0.036)**

Abbreviations: Cont, contraction; Exp, expiration; Insp, inspiration

** Student´s *t*-test for independent samples was performed.

^‡^ Mann-Whitney *U* test was utilized.

## Discussion

This study examined and compared the thickness of the diaphragm and muscles of the abdominal wall during inspiration, expiration and contraction between middle-aged patients recently diagnosed with moderate Covid-19 versus healthy participants. No significant differences in the thickness of the EO, IO, TrA, RA muscles or the IRD were observed between those with and without a recent diagnosis of moderate Covid-19.

To the best of our knowledge, no other studies have assessed the thickness of the abdominal wall muscles in people with Covid-19. Formenti et al. developed an ultrasonography study in both parasternal intercostal and diaphragm muscles in severe cases of coronavirus disease [35]. Individuals who survived reported differences in echogenicity values for both parasternal intercostal and diaphragm muscle. Regarding the muscle architecture and according with the findings of the present study, no structural changes were showed in respiratory muscles between alive and dead individuals. Fantini et al. showed a correlation between diaphragm thickness evaluated with B-mode ultrasonography and global respiratory alterations [36] and several authors have employed B-mode ultrasonography to visualize the diaphragmatic excursion, which positively correlates with lung inspiratory volumes [37, 38]. Whittaker et al. reported an association between hypocapnia and increased modulation of TrA thickness as assessed by USI during rest. The central nervous system coordinates the control of the abdominal muscles in synergy with the diaphragm during postural and respiratory tasks [39]. This coordinated activity modulates abdominal pressures and contributes to spinal stability [40, 41]. Sonographic abnormalities have been observed for the diaphragm in post severe Covid-19 patients [23]. Specifically, patients who were hospitalized for severe Covid-19 showed a decrease in the muscle contraction capacity of the diaphragm with respect to a non-Covid-19 cohort as measured by the thickening ratio (muscle thickness at maximal inspiration/end-expiration) quantified by USI. These findings likely reflect the negative impact of hospitalization and inactivity resulting in muscle atrophy. In contrast, the current study demonstrated no difference of the thickness of the diaphragm in people with moderate Covid-19 who did not require hospitalization. Whilst speculative, this could imply that a healthy diaphragm could be a protective factor against severe Covid-19 [22]. Despite non-significant results were found for the thickness of the abdominal wall and diaphragm muscles, the assessment and management of these muscles should be considered due clinical implications in the respiratory system.

### Methodological considerations

Given that this is a pilot study with a small sample size, the results should be interpreted with caution. Further important consideration is that the ultrasonography examiner was not blinded. Respiratory disturbances such as dyspnea, respiratory muscle strength, or cardiorespiratory function were not assessed in this study. Due to the certain restrictions related to the Covid-19 pandemic, a patient cardiopulmonary and fatigue evaluation was not available. Morphological adaptations typically take time to develop and it may be that our participants were tested too soon after the onset of the Covid-19 infection to detect changes in muscle morphology. Testing those with long Covid with ongoing respiratory dysfunction may likely yield different results.

Future studies examining the abdominal muscles are needed in severe post-Covid-19 patients, to determine whether the abdominal muscles are affected as a physical sequela of hospitalization. In addition, an assessment with ultrasonography M-mode could be useful in future studies to allow dynamic muscle evaluation regarding the contraction time, velocity, and time variables. Additional variables such as contraction ratio or peri-muscular connective tissue assessment could also be considered in future studies.

## Conclusion

This study revealed that the thickness of TrA, IO, EO, RA and the diaphragm as well as the IRD is not modified in people diagnosed but not hospitalized with a recent moderate Covid-19 infection.
